# Effects of small-sided games training on physical performance in youth team-sport athletes: a systematic review and meta-analysis

**DOI:** 10.3389/fphys.2026.1803471

**Published:** 2026-04-07

**Authors:** Yang Liu, Fengming Zhang, Shanshan Kong, Mykola Bezmylov, Ertao Yan

**Affiliations:** 1 National University of Ukraine on Physical Education and Sport, Kyiv, Ukraine; 2 Jilin Normal University, Siping, China; 3 Shandong Jiaotong University, Jinan, China

**Keywords:** physical performance, small-sided games, team sports, training intervention, youth athletes

## Abstract

**Objectives:**

This study aimed to investigate the effects of small-sided games (SSG) on physical performance in youth team-sport athletes.

**Methods:**

Four databases (Web of Science Core Collection, PubMed, Scopus and Embase) were searched. Methodological quality and certainty of evidence were assessed using the Cochrane Risk of Bias 2 (RoB 2) tool and the Grading of Recommendations Assessment, Development, and Evaluation (GRADE) approach, with the review conducted and reported in accordance with the PRISMA 2020 guidelines. All statistical analyses and meta-analyses were conducted using STATA 18.0.

**Results:**

A total of 15 studies involving 494 participants were included. Compared with control groups, SSG significantly improved maximal aerobic capacity (SMD = 0.78, 95% CI: 0.30 to 1.27, p = 0.001), intermittent high-intensity endurance (SMD = 1.05, 95% CI: 0.70 to 1.40, p< 0.001), sprint acceleration (SMD = −0.55, 95% CI: −1.02 to −0.09, p = 0.019), change-of-direction ability (SMD = −0.85, 95% CI: −1.38 to −0.33, p = 0.001), and lower-limb explosive power (SMD = 0.60, 95% CI: 0.25 to 0.96, p = 0.001), whereas no statistically significant improvement was observed for maximal sprint speed (SMD = −0.30, 95% CI: −0.70 to 0.10, p = 0.142).

**Conclusion:**

This systematic review and meta-analysis indicates that small-sided games training generally benefits physical performance in youth team-sport athletes. Consistent improvements were observed in intermittent high-intensity endurance, sprint acceleration, lower-limb explosive power, and change-of-direction ability, with additional benefits for maximal aerobic capacity, whereas effects on maximal sprint speed appear limited. Future studies should aim to refine SSG training protocols and clarify how specific training configurations influence maximal sprint speed and other match-related performance outcomes in youth team-sport athletes.

**Systematic Review Registration:**

https://www.crd.york.ac.uk/prospero/, identifier CRD420261295914.

## 1 Introduction

Small-sided games (SSG) training has attracted growing interest in team-sport physical training (Hammami et al., 2018a; Bredt et al., 2020; Fernández-Espínola et al., 2020; Mikalonytė et al., 2022; Clemente, 2025; Zhang et al., 2025). Coaches usually reduce player numbers and shrink or adjust the playing area, while keeping some match rules or task goals. This setup raises training density and load, and it makes practice closer to the physical demands of real matches (Sarmento et al., 2018; Fernández-Espínola et al., 2020). For example, in soccer, small-format games are used to manipulate space and player numbers to elicit intermittent high-intensity running; in basketball and handball, they are used to promote repeated high-intensity actions and decision-making under space constraints; in rugby, to adjust contact and running demands; and in volleyball, to combine frequent jumping actions with tactical constraints (Clemente et al., 2021; Zanin et al., 2021; de Oliveira Castro et al., 2022; Zhang et al., 2025). Compared with more traditional drill-based conditioning, SSG training tends to show higher action frequency and intermittent high-intensity patterns within sessions (Dellal et al., 2011; Hill-Haas et al., 2011; Arslan et al., 2020; Bredt et al., 2020; Ouertatani et al., 2022). Athletes must repeatedly perform running, sprinting, changes of direction and jumping in a continuously contested and changing setting, thereby stimulating both aerobic and anaerobic metabolism while also challenging neuromuscular function (Dellal et al., 2011; Hill-Haas et al., 2011; Selmi et al., 2020; Clemente et al., 2021). From a physiological perspective, research finds that SSG training can produce high heart-rate responses and considerable internal load, and that under some conditions the cardiovascular stimulus in SSG training matches that of high-intensity interval training (Ouertatani et al., 2022). Because SSG training includes varied actions and frequent load changes, it may also stimulate linear sprint performance, change-of-direction ability, lower-limb explosive power and cardiovascular endurance at the same time. This feature could help promote the coordinated development of several physical performance indicators (Los Arcos et al., 2015; Arslan et al., 2020; Mikalonytė et al., 2022; Nayıroğlu et al., 2022; Clemente et al., 2023). Still, the evidence is mixed when studies compare SSG training with conventional sport-specific training (Sarmento et al., 2018; Clemente et al., 2021). Different trials report different outcomes for cardiovascular endurance, linear sprint performance, change-of-direction ability and lower-limb explosive power. However, findings remain inconsistent when SSG training is compared with conventional sport-specific training in youth team-sport athletes.

These training effects are relevant because team sports impose complex physical demands. Team sports, such as soccer and basketball, show typical intermittent high-intensity activity patterns (Bishop and Girard, 2013). In matches, team-sport athletes repeatedly perform near-maximal actions over long periods; these actions include acceleration, deceleration, changes of direction, sprinting and jumping, and recovery between efforts is usually short (Bishop and Girard, 2013). Because match situations are complex and change frequently, team sports place wide physical demands on athletes, and performance therefore relies on the coordinated contribution of multiple physical capacities rather than on a single capacity (Farley et al., 2020). Research points to cardiovascular endurance, linear sprint performance, change-of-direction ability and lower-limb explosive power as important contributors to team sports outcomes (Bishop and Girard, 2013; Taylor et al., 2017; Harper et al., 2019; Spyrou et al., 2020; Thieschäfer and Büsch, 2022). Technical and tactical skills remain central in team sports, but good sport-specific physical fitness still provides the physiological base for high-quality technical execution and sound tactical decisions (Bishop and Girard, 2013; Davids et al., 2013; Taylor et al., 2017; Hammami et al., 2018b; Farley et al., 2020). Team-sport competitions are usually played at fairly high average intensities and include many short, high-intensity efforts, meaning that both aerobic and anaerobic energy systems contribute to match performance (Stolen et al., 2005; Harper et al., 2019; Fernández-Espínola et al., 2020; Mason et al., 2020). In youth, physical capacities grow quickly and stay highly plastic, so young athletes respond well to different training stimuli (Matos and Winsley, 2007; Thieschäfer and Büsch, 2022). For this reason, planned and systematic training helps improve current physical levels and also supports long-term development and future competitive potential in team-sport athletes (Granacher and Borde, 2017).

In youth team sports training practice, conventional sport-specific training remains the most common and foundational training approach, and it is mainly centered on sport-specific technical–tactical drills and routine team training, with training content closely aligned with match-related skills and contexts and a relatively stable overall structure, which allows athletes to consistently develop sport-specific technique and maintain basic sport-specific physical fitness (Silva et al., 2020; Formenti et al., 2021). However, this type of training usually places emphasis on the repeated reinforcement of technical–tactical objectives, and it provides limited stimulation for the highly variable and intermittent high-intensity demands that characterize competitive match play (Pinder et al., 2011). As a result, this training approach may be insufficient for simultaneously promoting the integrated development of multidimensional physical capacities and sport-specific performance abilities (Pinder et al., 2011; Clemente et al., 2021).

Although several systematic reviews and meta-analyses have looked at SSG training effects on physical performance in the last ten years, important gaps remain. Most reviews focus on adult athletes or on a single sport, especially football (Moran et al., 2019; Clemente et al., 2021; Gómez-Álvarez et al., 2024). As a result, direct evidence for youth team-sport athletes is limited. Many studies also pick physical outcome measures that cover only one physical domain, for example, aerobic capacity or change-of-direction ability, and thus they do not give a full picture of multidimensional physical performance (Moran et al., 2019; Clemente et al., 2023; Neag et al., 2024). In terms of comparisons, some reviews put SSG training against other active training types (Clemente et al., 2021a, 2021; Niu et al., 2025; Trotta et al., 2025). Few, however, systematically compare SSG training with the conventional sport-specific training methods that are commonly used in youth training practice. Therefore, the evidence base has not yet been assembled in a way that clarifies how SSG training actually affects the multidimensional physical performance development of youth team-sport athletes.

In contrast to prior meta-analyses on SSG training, the present study focuses specifically on youth team-sport athletes and compares SSG training with conventional sport-specific training methods that are commonly used in training practice. In addition, the present study examines differences across several physical performance outcomes. The analysis also considers key condition variables, including age, competitive level, total training sessions, and effective training time, and it further assesses the quality of evidence in the included studies.

This study aims to bring together current evidence on how SSG training affects physical performance in youth team-sport athletes by using a systematic review and meta-analysis.

## 2 Methods

This systematic review was conducted in accordance with the PRISMA 2020 statement, an updated guideline for reporting systematic reviews and meta-analyses to ensure transparency and completeness (Page et al., 2021), and the Cochrane Handbook for Systematic Reviews of Interventions (Higgins and Green, 2008).

### 2.1 Eligibility criteria

We used the PICOS framework, which lists Population, Intervention, Comparison, Outcome, and Study Design (Liberati et al., 2009). We included original peer-reviewed articles published in English up to 1 October 2025. We operationalized “youth” using chronological age because most studies report participant age rather than official competitive categories (e.g., U14–U16), which are not consistently provided across sports and settings. **Table 1** shows the full inclusion and exclusion criteria used in this study.

**Table 1:** Eligibility criteria.

Category	Inclusion criteria	Exclusion criteria
Population	Healthy youth team-sport athletes aged 10–19 years, competing at a minimum competitive level of Tier 2 (trained/developmental) (McKay et al., 2021), including basketball, soccer (football), handball, volleyball, and rugby players.	Athletes aged<10 or >19 years; non–team-sport athletes; individual sport athletes; recreationally active or non-athlete populations; mixed-age samples where data for athletes aged 10–19 years could not be extracted separately.
Intervention	Small-sided games (SSG) implemented as the primary training intervention, with an intervention duration of ≥2 weeks.	Interventions other than SSG (e.g., high-intensity interval training, resistance training, plyometric training, sprint interval training); comparisons between different SSG formats (SSG vs. SSG); acute or short-term SSG protocols (<2 weeks).
Comparison	Active control groups receiving conventional sport-specific training, including: (1) technical–tactical training; (2) regular team training; (3) sport-specific aerobic or interval training.	Other interventions or negative control groups.
Outcome	At least one measure of physical performance assessed before and after the intervention, including: Aerobic capacity: Final velocity completed at 30–15 intermittent fitness test (30–15 IFT) (km/h), Maximal oxygen uptake (VO_2max_) (ml/kg/min); (1) Linear sprint performance: 5–30 m sprint time (s); (2) Change-of-direction ability: CODS Time (s); (3) Lower-limb explosive power: CMJ Height (cm); (4) Intermittent high-intensity endurance: YYIRT-L1 Distance covered (m).	Outcomes unrelated to physical performance (e.g., injury rates, technical–tactical indices only, psychological variables); physiological markers without direct performance relevance (e.g., blood lactate, heart rate only); outcomes reported without sufficient data for effect size calculation (missing mean, SD, or sample size).
Study Design	Randomized controlled trials (RCTs).	Non-randomized studies, cross-sectional studies, case studies, and observational studies.

### 2.2 Information sources and search strategy

We searched Web of Science Core Collection, PubMed, Scopus, and Embase for relevant studies from database inception to 1 October 2025. We built the search strategy using free-text keywords. The search itself did not apply any restrictions on country or language, whereas the screening step included only studies published in English. The search focused on SSG and team sports and used the following main string: (“small-sided game*” OR “medium-sided game*” OR “large-sided game*” OR SSG OR SSGs OR “drill-based game*” OR “conditioned game*”) AND (basketball OR soccer OR football OR handball OR volleyball OR rugby OR “team sport*”). **Table 2** shows the complete search strategies for each database and the number of records retrieved.

**Table 2:** Complete search strategies across databases.

Database	Complete search strategy	Results
Web of Science Core Collection	TS = (“small-sided game*” OR “medium-sided game*” OR “large-sided game*” OR SSG OR SSGs OR “drill-based game*” OR “conditioned game*”) AND TS =(basketball OR soccer OR football OR handball OR volleyball OR rugby OR “team sport*”)	1510
PubMed	(“small-sided game”[Title/Abstract] OR “medium-sided game”[Title/Abstract] OR “large-sided game”[Title/Abstract] OR SSG[Title/Abstract] OR SSGs[Title/Abstract] OR “drill-based game”[Title/Abstract] OR “conditioned game”[Title/Abstract]) AND (basketball[Title/Abstract] OR soccer[Title/Abstract] OR football[Title/Abstract] OR handball[Title/Abstract] OR volleyball[Title/Abstract] OR rugby[Title/Abstract] OR “team sport”[Title/Abstract])	507
Scopus	TITLE-ABS-KEY((“small-sided game*” OR “medium-sided game*” OR “large-sided game*” OR SSG OR SSGs OR “drill-based game*” OR “conditioned game*”)AND (basketball OR soccer OR football OR handball OR volleyball OR rugby OR “team sport*”))	1374
Embase	(“small-sided game*”:ti,ab OR “medium-sided game*”:ti,ab OR “large-sided game*”:ti,ab OR SSG:ti,ab OR SSGs:ti,ab OR “drill-based game*”:ti,ab OR “conditioned game*”:ti,ab) AND (basketball:ti,ab OR soccer:ti,ab OR football:ti,ab OR handball:ti,ab OR volleyball:ti,ab OR rugby:ti,ab OR “team sport*”:ti,ab)	626

### 2.3 Study selection

We imported all records identified through database searches into EndNote (Clarivate Analytics) to manage references. Two authors (YL and FZ) removed duplicates by using both automated tools and manual checks. After this step, the same authors independently screened titles and abstracts to decide whether each study met the predefined inclusion criteria. For records that appeared eligible, the authors retrieved the full texts and independently reviewed them to confirm final inclusion.

If the two reviewers disagreed at any stage, they addressed the issue through discussion and by checking the original articles again. When disagreement remained, a third author (EY) made the final decision. The whole screening process followed the PRISMA 2020 guidelines.

### 2.4 Data extraction

We extracted data using pre-designed and standardized tables for study characteristics and intervention characteristics. Two authors (YL and FZ) completed the data extraction independently. The study characteristics table included author and publication year, sport, study group size and allocation, age (years, mean ± SD), competitive level, sex, randomization, control intervention, study design, physical performance tests, and the related outcomes. The intervention characteristics table included author and publication year, sport, intervention duration (weeks), sessions per week, total number of sessions, SSG formats, pitch dimensions, area per player, number of sets, number of repetitions, work duration, and rest intervals between sets and repetitions. A third author (EY) reviewed the extracted data to ensure consistency. We organized and managed all data using Microsoft Excel. When studies reported missing or unclear information, the authors contacted the original researchers by email or through ResearchGate. If no reply was obtained, the analyses relied on the available data, and this issue was noted when interpreting the results.

### 2.5 Outcome measures

We grouped cardiovascular-related outcomes under cardiorespiratory endurance, which covered maximal aerobic capacity and intermittent high-intensity endurance. We assessed maximal aerobic capacity using directly measured VO_2_max from laboratory tests and aerobic capacity values estimated from field-based tests, including the Yo-Yo intermittent recovery test level 1, 20 m shuttle run test, and 30–15 intermittent fitness test. We assessed intermittent high-intensity endurance using the distance completed in the Yo-Yo intermittent recovery test level 1. When a study used multiple tests to assess change-of-direction ability, we selected the most representative standardized test and included a single outcome per study to maintain data independence. We classified sprint-related outcomes as linear sprint performance. These outcomes included sprint acceleration performance, measured with 10 m and 20 m sprint tests, and maximal sprint speed performance, measured with the 30 m sprint test.

### 2.6 Risk of bias and certainty of evidence

We evaluated the risk of bias of the included randomized controlled trials at the study level using the Cochrane Risk of Bias 2 (RoB 2) tool, and we followed the guidance described by Sterne et al. (2019). The assessment covered five areas: the randomization process, deviations from intended interventions, missing outcome data, measurement of the outcome, and selection of the reported results. For each domain and for each study overall, we judged the risk of bias as low risk, as having some concerns, or high risk. Two authors (YL and FZ) completed the assessments independently, and they resolved disagreements through discussion. When agreement could not be reached, a third author (EY) made the final decision.

We then assessed the certainty of evidence for each physical performance outcome using the Grading of Recommendations Assessment, Development, and Evaluation (GRADE) approach (Goldet and Howick, 2013). Two authors (YL and FZ) independently performed the GRADE assessment, and a third author (EY) reviewed the results.

### 2.7 Statistical analysis

We included outcomes in the meta-analysis only when at least three studies reported pre- and post-intervention data for the same physical performance outcome domain in both the intervention and control groups (Impellizzeri et al., 2006; Chaouachi et al., 2014). If outcome measures were not comparable across studies because they differed in measurement methods, measurement scales, or assessment targets, or if they did not belong to the same outcome domain, we did not pool them and described them narratively in the systematic review (Higgins and Green, 2008). In the statistical analysis, for studies that used different tests but addressed the same outcome domain, we computed standardized mean differences (SMD) as the effect size and carried out meta-analyses on those SMDs. We did not statistically combine test results that reflected different outcome domains.

We calculated effect sizes for each outcome domain by comparing changes from pre- to post-intervention between the intervention and control groups. When available, we extracted the mean change (post–pre) and the corresponding SD of change directly. If the SD of change was not reported but pre-intervention and post-intervention SDs were available, we derived the SD of change using the standard formula: $SD_{\Delta }=\sqrt{SD_{pre}^{2}+SD_{post}^{2}-2r\cdot SD_{pre}\cdot SD_{post}}$, where r denotes the within-group pre–post correlation. Because r was rarely reported, we assumed r = 0.50 for the primary analyses. If required information was not available, we contacted study authors for clarification or missing data; where feasible, dispersion estimates were also derived from other reported statistics (e.g., SE, CI, or p values). Outcomes for which the required data could not be obtained were not included in the corresponding pooled analysis. Effect sizes were expressed as Hedges’ g and reported the associated 95% confidence intervals (95% CI) (Higgins and Green, 2008). We interpreted effect size magnitudes using established thresholds: trivial effect (<0.2), small effect (0.2–0.6), moderate effect (0.6–1.2), large effect (1.2–2.0), very large effect (2.0–4.0), and extremely large effect (>4.0) (Hopkins et al., 2009; Clemente et al., 2021a).

We used a random-effects model to pool results because the included studies differed in participant characteristics, intervention protocols, and measurement methods (Akdoğan et al., 2021). We evaluated heterogeneity across studies using the I² statistic. Values of 0 ≤ I²< 25% reflected low heterogeneity, 25% ≤ I²< 75% reflected moderate heterogeneity, and I² ≥ 75% reflected high heterogeneity (Higgins et al., 2003; Zaharia et al., 2023; Xu et al., 2024).

When a study included more than one eligible intervention arm that shared a single control group, we split the control-group sample size (N) across comparisons to avoid double-counting (Higgins and Green, 2008). For continuous outcomes, the control-group mean and SD were retained and only the control-group N was divided equally across eligible intervention arms, preventing inflated precision from repeated use of the same control participants. If a study did not report all required data, we contacted the authors for missing information. Outcomes without available data after these attempts were not included in the meta-analysis (Impellizzeri et al., 2006; Higgins and Green, 2008).

We ran subgroup analyses with a random-effects model and used p< 0.05 as the significance level (Akdoğan et al., 2021). We performed all analyses in Stata (version 18.0; StataCorp LLC, College Station, TX, USA). We predefined subgroup analyses to explore heterogeneity in SSG effects, using age, competitive level, total training sessions, and effective training time as subgroup factors (Higgins and Green, 2008). We split continuous variables (age, total training sessions, effective training time) at the median of the studies that provided the relevant outcome data (Impellizzeri et al., 2006). We assigned categorical variables (for example, competitive level) according to the descriptions in the original studies. To reduce misleading heterogeneity, we calculated medians only from studies that supplied the data for the specific outcome being analyzed, rather than from the full set of included studies. Subgroup analyses were exploratory, and their results should be interpreted cautiously because of limited numbers of studies per subgroup and the use of study-level factors.

Initially, we intended to assess publication bias using funnel plots and Egger’s test when appropriate; however, because fewer than 10 studies were available for any outcome, these methods were not used, as they are underpowered and may yield misleading conclusions.

## 3 Results

### 3.1 Study selection

**Figure 1:**
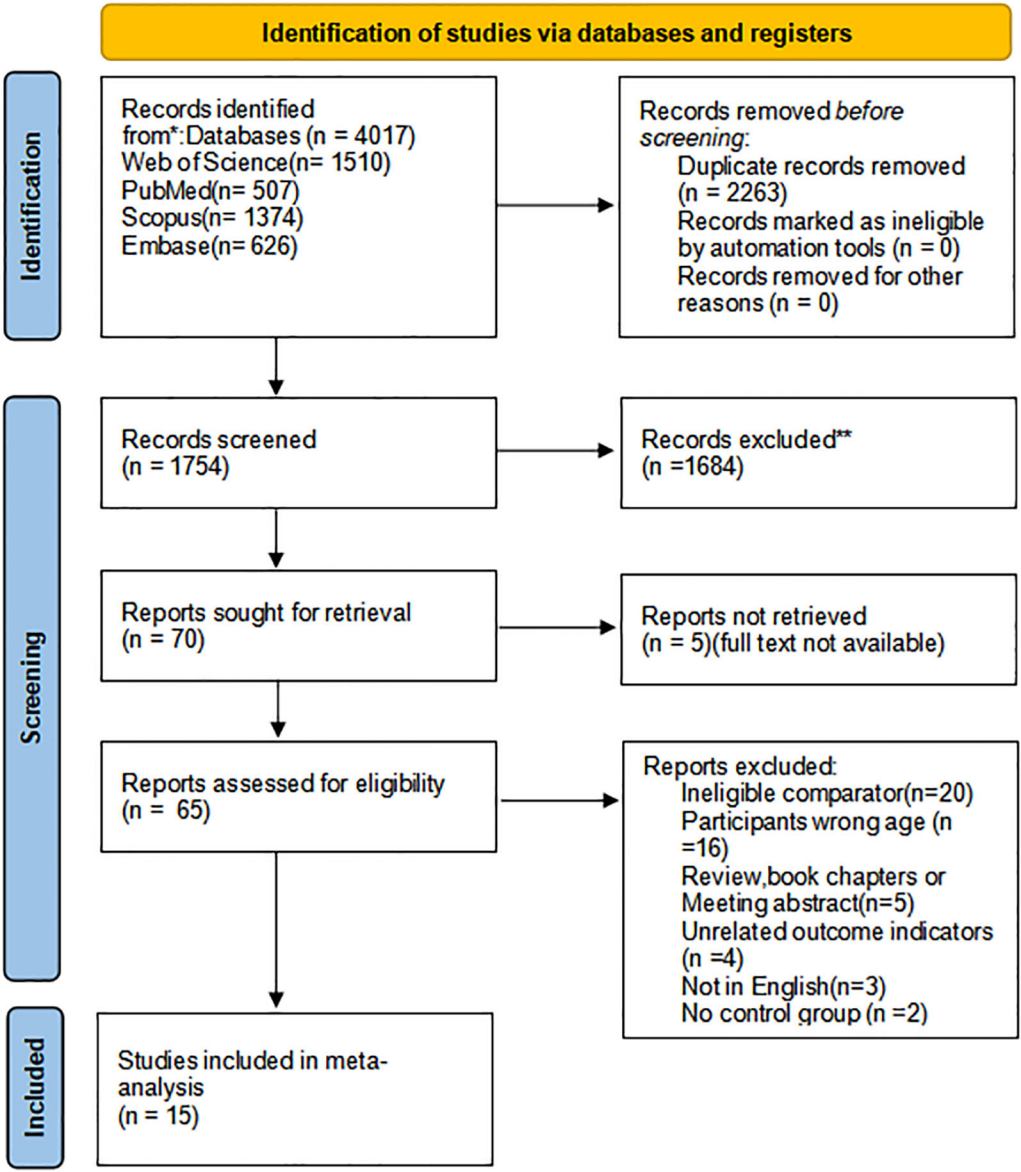
Systematic review search and screening procedure.

**Figure 1** presents the study selection process. We identified 4,017 records through database searching, including Web of Science Core Collection (n = 1,510), PubMed (n = 507), Scopus (n = 1,374), and Embase (n = 626). After we removed duplicates with EndNote (n = 2,263), 1,754 records remained for screening. After title and abstract screening, 70 records were retained. We then excluded five records because the full texts were not available, and 65 full-text articles underwent eligibility assessment. We excluded 50 articles for the following reasons: ineligible comparator (n = 20), participants outside the target age range (n = 16), review articles, book chapters, or meeting abstracts (n = 5), unrelated outcome indicators (n = 4), not in English (n = 3), and no control group (n = 2). Finally, 15 studies met the inclusion criteria and were included in the meta-analysis (Impellizzeri et al., 2006; Chaouachi et al., 2014; Akdoğan et al., 2021; Jurisic et al., 2023; Zaharia et al., 2023; Pratama et al., 2024; Wang et al., 2024; Wen et al., 2024; Xu et al., 2024; Li et al., 2025; Majkić et al., 2025; Neag et al., 2025; Prasad et al., 2025; Wen et al., 2025; Yang et al., 2025).

### 3.2 Study characteristics

**Table 3** presents the participant characteristics of the 15 included studies.

**Table 3:** Characteristics of studies included in the meta-analysis.

Study	Sport	Study group (n)	Age (years) mean ± SD	Competitive level	Sex	Randomized	Control intervention	Study design	Tests	Outcomes
Yang et al. (2025)	Soccer	SSG: 15 CG: 15	SSG: 15.7 ± 0.6 CG: 15.7 ± 0.5	Tier 2	Female	Yes	Regular soccer training	Parallel	30–15 IFT 30-m Linear sprint test CMJ test	Final velocity completed at 30–15 IFT (km/h) 30-m Sprint time (s) CMJ Height (cm)
Prasad et al. (2025)	Soccer	SSG: 9 CG: 9	SSG: 15.44 ± 1.01 CG: 15.77 ± 0.97	Tier 2	Female	Yes	Regular soccer training	Parallel	Illinois agility test	CODS Time (s)
Neag et al. (2025)	Soccer	SSG: 16 CG: 15	SSG: 10.63 ± 0.48 CG: 10.89 ± 0.31	Tier 2	Male	Yes	Regular soccer training	Parallel	10-m Linear sprint test 20-m Linear sprint test 5–0–5 Test	10-m Sprint time (s) 20-m Sprint time (s) CODS Time (s)
Wen et al. (2025)	Soccer	SSGlBT: 16 SSGfBT: 16 CG: 15	SSGlBT: 16.6 ± 0.8 SSGfBT: 16.6 ± 0.6 CG: 16.6 ± 0.5	Tier 2	Male	Yes	Regular soccer training	Parallel	10-m Linear sprint test 30-m Linear sprint test YYIRT-L1	10-m Sprint time (s) 30-m Sprint time (s) YYIRT-L1 Distance covered (m)
Majkić et al. (2025)	Soccer	SSG: 11 CG: 11	SSG: 16.11 ± 0.41 CG: 16.11 ± 0.41	Tier 3	Male	Yes	Regular soccer training	Parallel	CMJ 10-m Linear sprint test 30-m Linear sprint test T-test	CMJ Height (cm) 10-m Sprint time (s) 30-m Sprint time (s) CODS Time (s)
Li et al. (2025)	Basketball	SSGfreeD: 15 SSGlimitD: 15 CG: 16	SSGfreeD: 15.7 ± 0.6 SSGlimitD: 15.7 ± 0.5 CG: 15.8 ± 0.5	Tier 2	Male	Yes	Regular basketball training	Parallel	YYIRT-L1	YYIRT-L1 Distance covered (m)
Xu et al. (2024)	Basketball	SSG: 10 CG: 10	SSG: 16.9 ± 0.3 CG: 16.2 ± 0.6	Tier 3	Male	Yes	Regular basketball-specific technical and tactical drills	Parallel	20-m Linear sprint test GXT 5–0–5 Test CMJ Test	20-m Sprint time (s) VO_2_max (mL/kg/min) CODS Time (s) CMJ Height (cm)
Wen et al. (2024)	Soccer	SSG: 16 CG: 16	SSG: 17.2 ± 1.2 CG: 17.0 ± 1.1	Tier 2	Female	Yes	Regular soccer training	Parallel	30–15 IFT CMJ Test	Final velocity completed at 30–15 IFT (km/h) CMJ Height (cm)
Wang et al. (2024)	Soccer	SSGlw2: 16 SSGlw1: 16 CG: 16	Overall: 16.4 ± 0.6	Tier 2	Male	Yes	Regular soccer training	Parallel	30-m Linear sprint test YYIRT-L1	30-m Sprint time (s) YYIRT-L1 Distance covered (m)
Pratama et al. (2024)	Soccer	SSG: 23 CG: 23	Overall: 15–17	Tier 2	ND	Yes	Regular soccer training	Parallel	YYIRT-L1	VO_2_max (mL/kg/min)
Jurisic et al. (2023)	Handball	SSG: 15 CG: 15	SSG: 16.20 ± 0.94 CG: 16.00 ± 0.93	Tier 2	Female	Yes	Regular handball training	Parallel	T-test	CODS Time (s)
Zaharia et al. (2023)	Soccer	SSG: 20 CG: 20	SSG: 17.49 ± 0.61 CG: 17.66 ± 0.54	Tier 3	Male	Yes	Soccer-specific aerobic endurance training	Parallel	20-m MSFT 10-m Linear sprint test 20-m Linear sprint test Illinois agility test	VO_2_max (mL/kg/min) 10-m Sprint time (s) 20-m Sprint time (s) CODS Time (s)
Akdoğan et al. (2021)	Soccer	SSG: 11 CG: 9	Overall: 14.6 ± 0.5	Tier 3	Male	Yes	Low-intensity soccer technical and tactical training	Parallel	10-m Linear sprint test 30-m Linear sprint test YYIRT-L1 RAST	10-m Sprint time (s) 30-m Sprint time (s) YYIRT-L1 Distance covered (m) RAST Total time (s)
Chaouachi et al. (2014)	Soccer	SSG: 12 CG: 12	Overall: 14.2 ± 0.9	Tier 3	Male	Yes	Regular soccer training	Parallel	10-m Linear sprint test 30-m Linear sprint test 10–8–8–10 m Test CMJA Test	10-m Sprint time (s) 30-m Sprint time (s) CODS Time (s) CMJA Height (cm)
Impellizzeri et al. (2006)	Soccer	SSG: 20 CG: 20	Overall: 17.2 ± 0.8	Tier 3	Male	Yes	Generic aerobic interval training	Parallel	GXT	VO_2_max (mL/kg/min)

SSG, small-sided games; CG, control group; Tier 2, trained/developmental; Tier 3, highly trained/national level; 30–15 IFT, 30–15 intermittent fitness test; CMJ, countermovement jump; YYIRT-L1, Yo-Yo intermittent recovery test level 1; 5–0–5 test, 505 change-of-direction test; GTX, graded exercise test; T-test, T-test of agility; 20-m MSFT, 20-m multistage fitness test(beep test); CMJA, countermovement jump with arm swing; SSGlBT, small-sided games with limited ball touches; SSGfBT, small-sided games with free ball touches; SSGlw2, small-sided games with a length-to-width ratio of 2.0; SSGlw1, small-sided games with a length-to-width ratio of 1.0; VO2max, Maximal oxygen uptake; SSGfreeD, small-sided games in free play; SSGlimitD, small-sided games with limited dribbling.

#### 3.2.1 Sex

Ten of the 15 studies involved male athletes, four studies focused on female athletes, and one study did not report sex.

#### 3.2.2 Age

Participants’ ages ranged from 10.76 to 17.58 years, and the overall mean age across studies was 15.89 years.

#### 3.2.3 Sample size

All included studies involved a total of 494 youth team-sport athletes. The number of participants differed between studies, with sample sizes ranging from 18 athletes (Prasad et al., 2025) to 48 athletes (Wang et al., 2024). The mean sample size across studies was 33 athletes. In terms of sport type, soccer players comprised 398 participants (80.6%), while basketball players accounted for 66 participants (13.4%) and handball players for 30 participants (6.1%).

#### 3.2.4 Competitive level

In terms of competitive level, nine studies included Tier 2 athletes and six studies included Tier 3 athletes (McKay et al., 2021).

#### 3.2.5 Study design

All studies used a randomized controlled trial design.

#### 3.2.6 Type of control intervention

For the type of control intervention, most studies relied on conventional sport-specific training. Only one study used soccer-specific aerobic endurance training (Zaharia et al., 2023), and one study applied generic aerobic interval training as the control condition (Impellizzeri et al., 2006).

#### 3.2.7 Outcome domains

Across outcome domains, six studies assessed cardiovascular endurance, seven assessed sprint acceleration, six assessed maximal sprint speed performance, seven assessed change-of-direction ability, four assessed lower-limb explosive power, and four assessed intermittent high-intensity endurance.

#### 3.2.8 Testing methods

Across studies, physical performance outcomes were measured using established field- and laboratory-based tests. These tests targeted cardiorespiratory endurance, linear sprint performance, change-of-direction ability, and lower-limb explosive performance, with detailed test protocols and corresponding outcomes reported in **Table 3**.

#### 3.3 Methodological characteristics of the interventions

**Table 4** presents the methodological characteristics of the SSG intervention protocols. Intervention duration varied across studies. Most interventions lasted between 6 and 8 weeks, whereas one study reported a duration of 12 weeks (Impellizzeri et al., 2006) and another reported a duration of 18 weeks (Neag et al., 2025). Across studies, sessions per week ranged from 2 to 4. The SSG formats ranged from 1v1 to 6v6. Pitch dimensions ranged between 15 × 7 m (Li et al., 2025) and 40 × 50 m (Impellizzeri et al., 2006), while area per player ranged from 15 m² to 250 m². The structure of SSG training, including sets, reps, effective training time, and the duration of rest between sets and rest between reps, varied among studies.

**Table 4:** Descriptive characteristics of SSG intervention protocols.

Study	Sport	Intervention duration (weeks)	Sessions per week	Total sessions (n)	SSG formats	Pitch dimensions (m × m)	Area per player (m2)	Sets	Reps	Effective training time (min)	Rest between sets (min)	Rest between reps (min)
Yang et al. (2025)	Soccer	6	2	12	1v1 2v2 3v3 1v1 2v2 3v3	15 × 8 20 × 14 30 × 18 15 × 8 20 × 14 30 × 18	60 70 90 60 70 90	2 2 1 3 3 1	6 4 8 6 4 8	1 2 2 1 2 3	ND	2
Prasad et al. (2025)	Soccer	8	3	24	3v3 4v4 5v5 4v4 + 2	30 × 20	100 75 60 60	ND	ND	30–35 35–40 40–45 45–50	ND	ND
Neag et al. (2025)	Soccer	18	2	36	1v1 1v1+GK 2v1 2v1+GK 2v2 2v2+GK	15 × 20 15 × 20 15 × 20 15 × 20 15 × 25 15 × 25	150 150 100 100 93.75 93.75	2 2 1 2 2 2	4 5 6 4 4 5	0.10–0.15 0.10–0.15 0.12–0.17 0.12–0.17 0.13–0.18 0.13–0.18	3	1
Wen et al. (2025)	Soccer	6	3	18	4v4 3v3 5v5+GK 4v4 3v3 5v5+GK 4v4+GK 2v2 4v4+GK	35 × 35 30 × 20 40 × 30 35 × 35 30 × 20 40 × 30 40 × 30 25 × 15 40 × 30	153.13 100 120 153.13 100 120 150 93.75 150	3 4 3 4 5 4 4 6 4	ND	5 3 5 5 3 5 5 2 5	2	ND
Majkić et al. (2025)	Soccer	6	2	12	5v5 6v6	34 × 22 36 × 25	74.80 75	3	ND	3	3	ND
Li et al. (2025)	Basketball	8	2	16	3v3 2v2	15 × 7	17.50 26.25	4 6 8 5	ND	3 2 2 3	2	ND
Xu et al. (2024)	Basketball	6	3	18	3v3	28 × 7.5	35	3	ND	5	2	ND
Wen et al. (2024)	Soccer	8	2	16	2v2 4v4	25 × 16 35 × 23	100 100.63	4 4	ND	2 4	3	ND
Wang et al. (2024)	Soccer	8	2	16	4v4	40 × 20 28 × 28	100 98	3	ND	4	3	ND
Pratama et al. (2024)	Soccer	8	4	32	ND	ND	ND	ND	ND	ND	ND	ND
Jurisic et al. (2023)	Handball	8	2	16	3v3	20 × 20	66.70	ND	ND	≈45	ND	ND
Zaharia et al. (2023)	Soccer	8	2	16	3v3 4v4+GK	18 × 15 40 × 32	45 160	6 8	ND	1 2.5	0.5 2.5	ND
Akdoğan et al. (2021)	Soccer	6	2	12	4v4 3v3 2v2	25 × 32 20 × 30 16 × 25	100 100 100	4 4 4	ND	4 4 2	3	ND
Chaouachi et al. (2014)	Soccer	6	2	12	1v1 1v1 1v1 2v2 2v2 2v2 2v2 2v2 3v3 3v3	10 × 20 10 × 20 10 × 20 20 × 20 20 × 20 20 × 20 20 × 20 20 × 20 20 × 30 20 × 30	100	2 2 2 2 2 2 2 2 2 1	2 3 4 2 3 3 4 3 2 2	0.5 0.5 0.5 1 1 1 1 1 2 2	ND	2 2 2 2 1 1 1 2 2 2
Impellizzeri et al. (2006)	Soccer	12	2	24	3v3+GK 4v4+GK	25 × 35 40 × 50	145.83 250	4	ND	4	3	ND

SSG, small-sided games; GK, goal keeper; ND, not described.

### 3.4 Study risk of bias assessment

**Figure 2:**
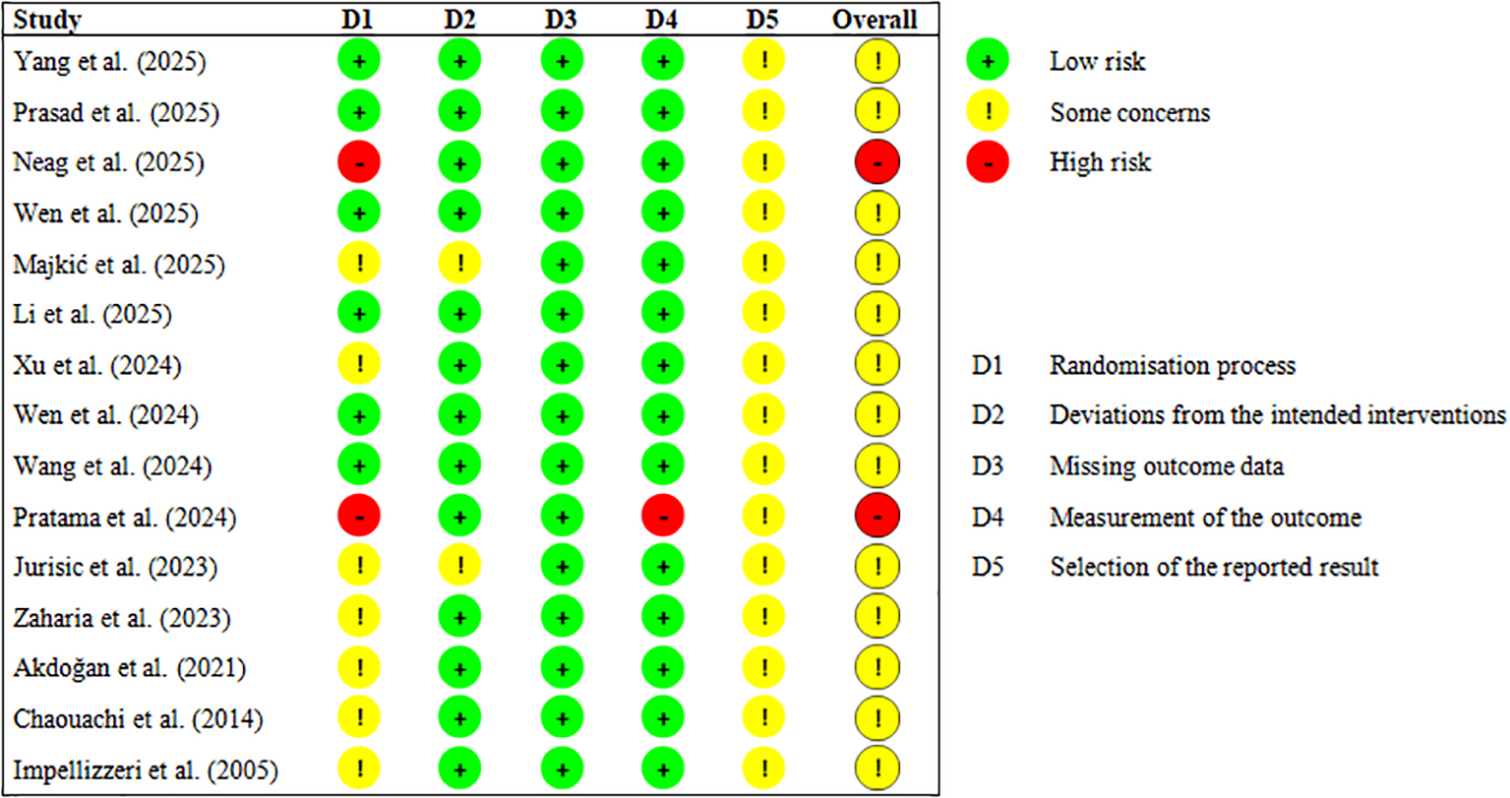
Risk of overall bias.

**Figure 3:**
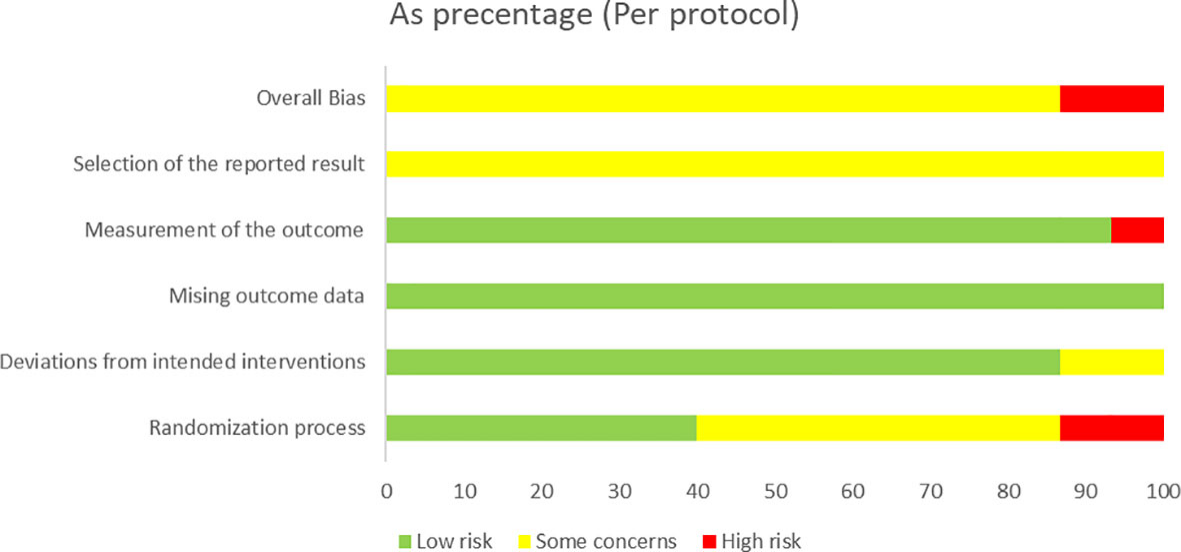
RoB-2 assessment.

The risk of bias assessments and the overall risk of bias judgments for the 15 included studies are shown in **Figures 2**, **Figure 3**. In general, most studies were rated as having some concerns for the overall risk of bias. These concerns mainly arose from issues related to the randomization process and the selection of the reported results.

### 3.5 Certainty of evidence

**Table 5** shows the GRADE assessment of evidence certainty. Most physical-performance outcomes had low certainty of evidence. The certainty for lower-limb explosive performance and for intermittent high-intensity endurance was moderate.

**Table 5:** GRADE summary of evidence.

Outcome	Study design	Number of participants	GRADE assessment					
			Risk of bias	Inconsistency	Indirectness	Imprecision	Publication bias	Certainty of evidence
Maximal aerobic capacity	6 RCTs	197	Not serious	Serious^b^	Not serious	Serious^d^	Undetected	Low
Sprint acceleration	7 RCTs	204	Not serious	Serious^b^	Not serious	Serious^d^	Undetected	Low
Maximal sprint speed	6 RCTs	191	Not serious	Serious^b^	Not serious	Serious^cd^	Undetected	Low
Change-of-direction ability	7 RCTs	185	Not serious	Serious^b^	Not serious	Serious^d^	Undetected	Low
Lower-limb explosive power	5 RCTs	128	Not serious	Not serious	Not serious	Serious^d^	Undetected	Moderate
Intermittent high-intensity endurance	4 RCTs	161	Not serious	Not serious	Not serious	Serious^d^	Undetected	Moderate

b: Serious inconsistency due to substantial statistical heterogeneity (I² > 50%).

c: The confidence intervals crossed the line of no effect, indicating uncertainty in the estimated effect.

d: The total sample size was below the optimal information size required to reliably detect a meaningful effect.

### 3.6 Main effects

#### 3.6.1 Cardiovascular endurance

**Figure 4:**
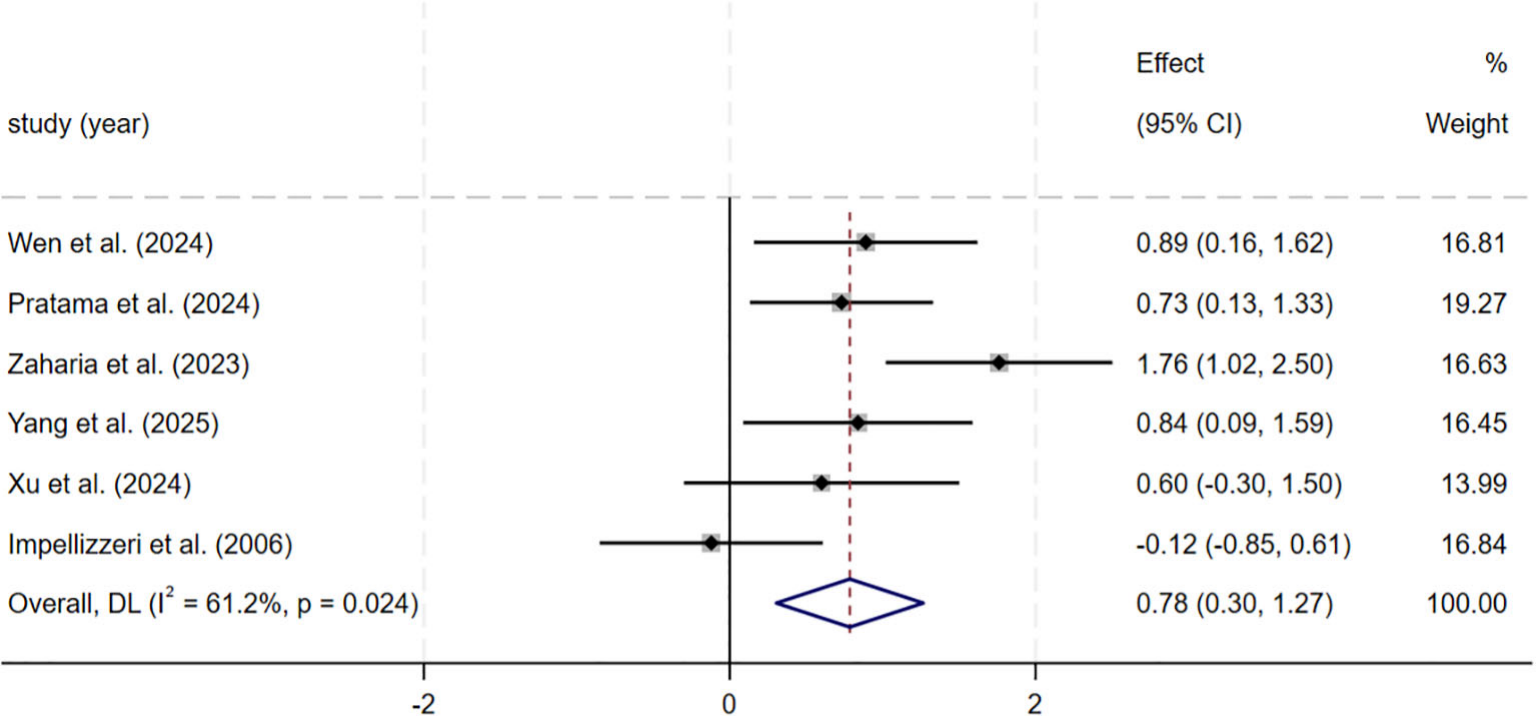
Forest plot of SSG on maximal aerobic capacity. Weights are from random-effects model.

For maximal aerobic capacity, the meta-analysis of six studies (n = 197) showed a moderate and statistically significant improvement after SSG training compared with control groups (SMD = 0.78, 95% CI: 0.30–1.27, p = 0.001). Heterogeneity was moderate (I² = 61.2%). This pooled estimate combined directly measured VO_2_max and aerobic capacity values estimated from field-based tests, which may contribute to construct/measurement heterogeneity. Sensitivity analysis using a leave-one-out approach showed that exclusion of Zaharia et al. (2023) led to a smaller pooled effect (SMD = 0.59, 95% CI: 0.23–0.96, p = 0.001) and a clear reduction in heterogeneity (I² = 18.8%). The effect direction and statistical significance did not change, indicating that the results were stable (**Figure 4**).

**Figure 5:**
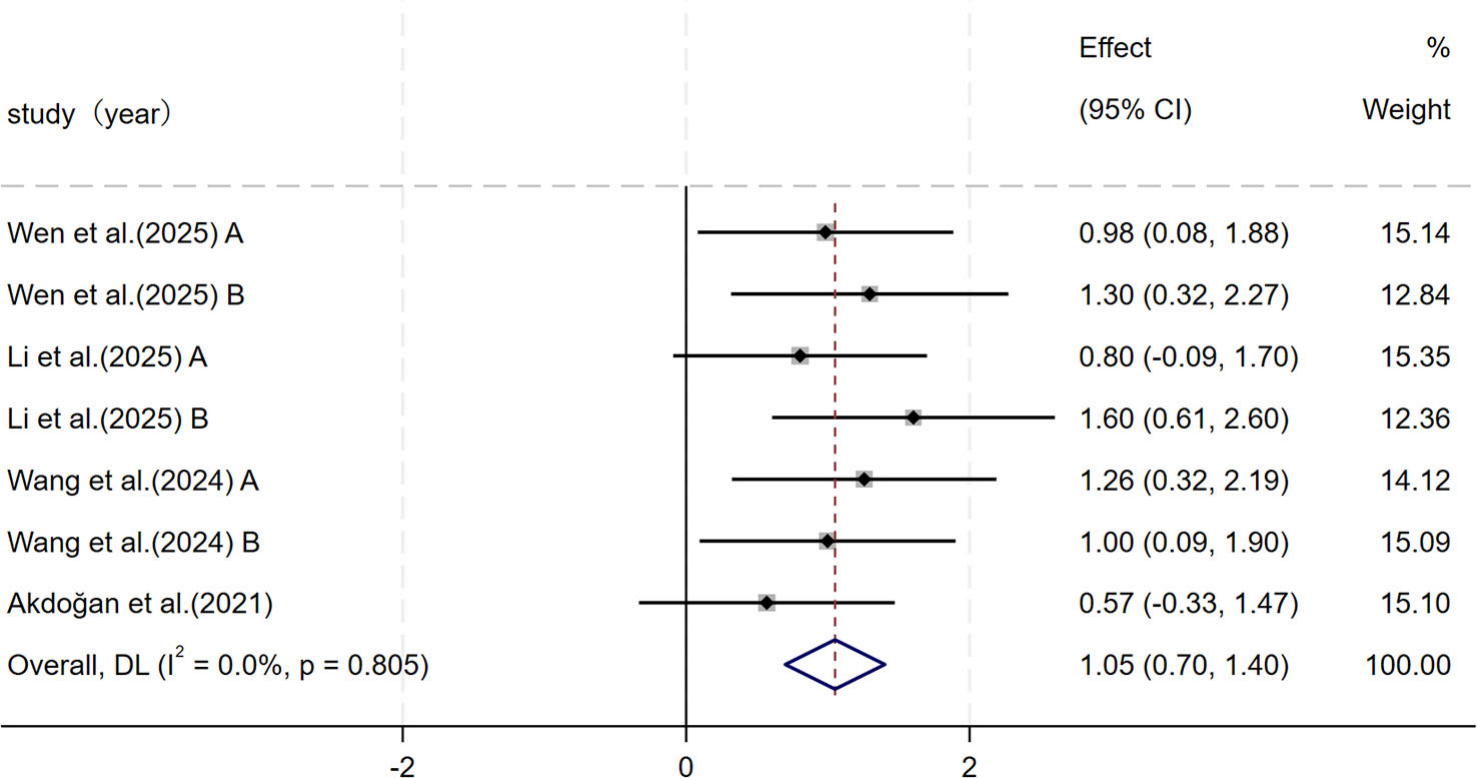
Forest plot of SSG on intermittent high-intensity endurance. Weights are from random-effects model.

For intermittent high-intensity endurance, the meta-analysis of four studies (n = 161) showed a moderate and significant improvement after SSG training compared with control conditions (SMD = 1.05, 95% CI: 0.70–1.40, p< 0.001). In this outcome, heterogeneity was low (I² = 0.0%) (**Figure 5**).

#### 3.6.2 Linear sprint performance

**Figure 6:**
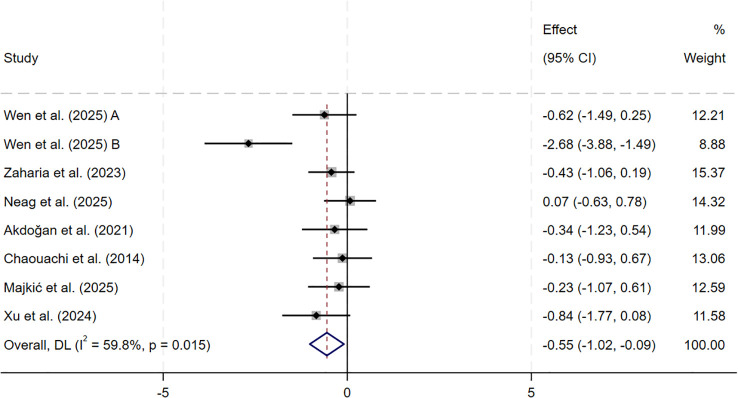
Forest plot of SSG on sprint acceleration. Weights are from random-effects model.

For sprint acceleration, the meta-analysis of seven studies (n = 204) showed a small but significant improvement after SSG training compared with control groups (SMD = −0.55, 95% CI: −1.02 to −0.09, p = 0.019). Heterogeneity was moderate (I² = 59.8%). Sensitivity analysis using a leave-one-out approach showed that exclusion of Wen et al. (2025) reduced the pooled effect (SMD = −0.33, 95% CI: −0.63 to −0.03, p = 0.03) and eliminated heterogeneity (I² = 0.0%). The effect direction and statistical significance did not change, indicating that the results were stable (**Figure 6**).

**Figure 7:**
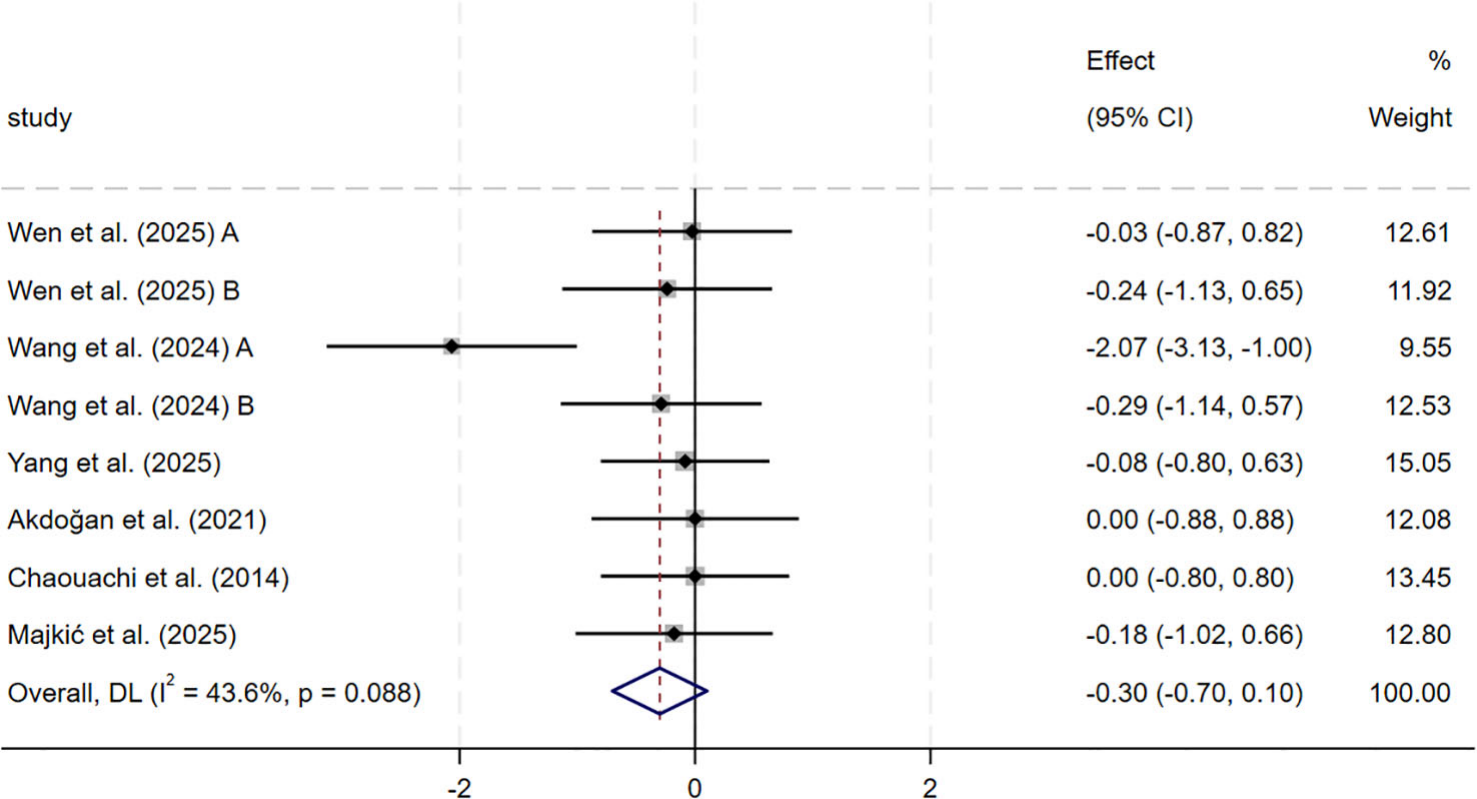
Forest plot of SSG on maximal sprint speed. Weights are from random-effects model.

For maximal sprint speed, the meta-analysis of six studies (n = 191) showed no statistically significant change after SSG training compared with control groups (SMD = −0.30, 95% CI: −0.70 to 0.10, p = 0.142). Heterogeneity was moderate (I² = 43.6%). Sensitivity analysis using a leave-one-out approach showed that exclusion of Wang et al. (2024) (A) further reduced the pooled effect (SMD = −0.11, 95% CI: −0.42 to 0.20, p = 0.482) and lowered heterogeneity to I² = 0.0%. The effect direction and statistical significance remained consistent, indicating that the results were stable (**Figure 7**).

#### 3.6.3 Change-of-direction ability

**Figure 8:**
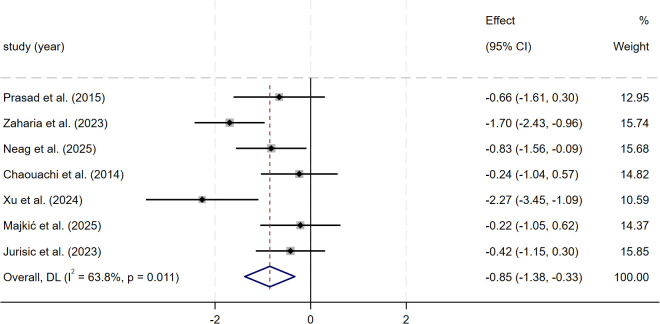
Forest plot of SSG on change-of-direction ability. Weights are from random-effects model.

For change-of-direction ability, the meta-analysis of seven studies (n = 185) showed a moderate and significant improvement after SSG training compared with control groups (SMD = −0.85, 95% CI: −1.38 to −0.33, p = 0.001). Heterogeneity was moderate (I² = 63.8%). Sensitivity analysis using a leave-one-out approach showed that heterogeneity did not change substantially after excluding individual studies. The effect direction and statistical significance remained consistent, indicating that the results were stable (**Figure 8**).

#### 3.6.4 Lower-limb explosive power

**Figure 9:**
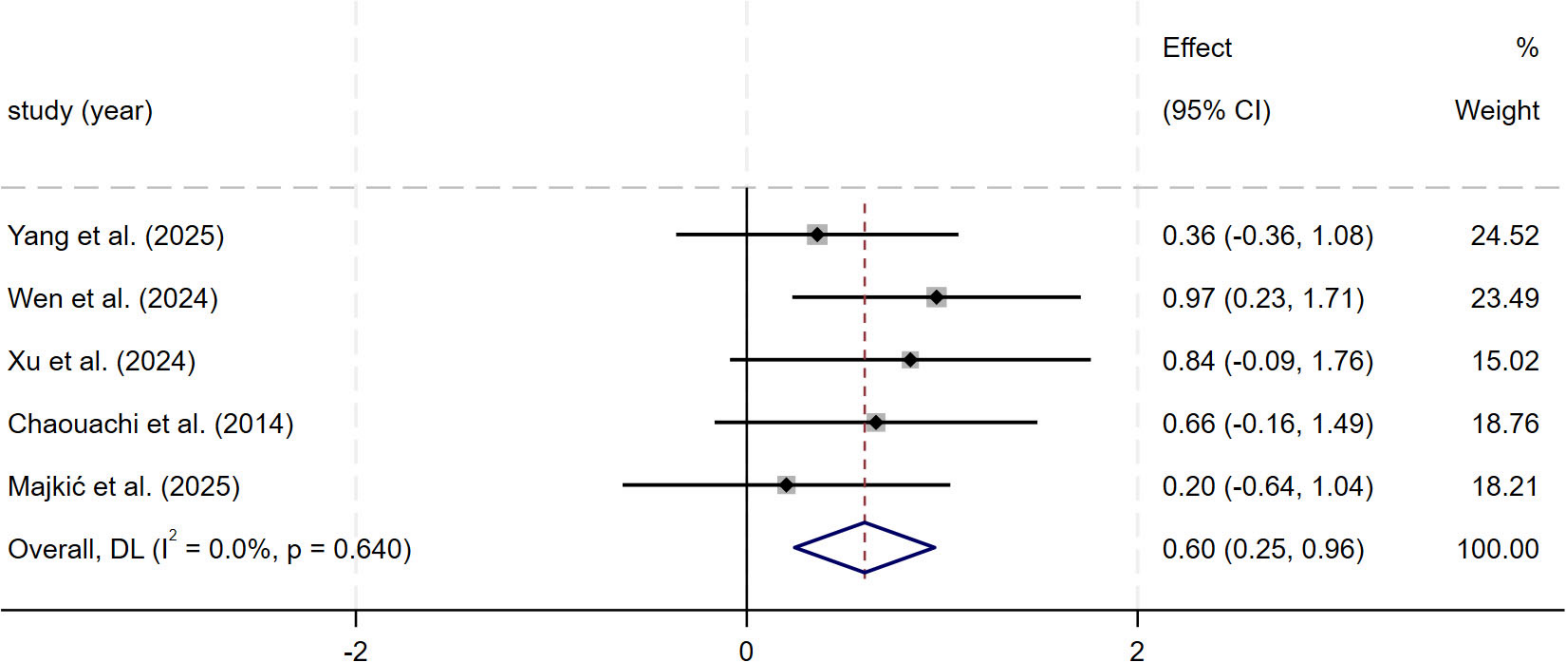
Forest plot of SSG on lower-limb explosive power. Weights are from random-effects model.

For lower-limb explosive power, the meta-analysis of five studies (n = 128) showed a moderate and significant improvement after SSG training compared with control groups (SMD = 0.60, 95% CI: 0.25–0.96, p = 0.001). In this outcome, heterogeneity was low (I² = 0.0%) (**Figure 9**).

### 3.7 Subgroup analyses

The prespecified subgroup analyses showed that, for all physical performance outcomes, including maximal aerobic capacity and sprint acceleration, no statistically significant differences were identified between subgroups when the studies were classified by age, competitive level, total training sessions, or effective training time (all P for subgroup difference > 0.05). These results indicated that, within the available evidence, these training characteristics did not act as effect modifiers of SSG training. The intervention effects were similar in magnitude and direction across all examined subgroups. Detailed subgroup analysis results are presented in **Table 1**.

## 4 Discussion

We conducted a systematic review and meta-analysis to assess the effects of SSG training on the physical performance of youth team-sport athletes. Fifteen randomized controlled trials met our inclusion criteria, totaling 494 athletes, of whom 272 received SSG training and 222 received conventional sport-specific training. Compared with conventional sport-specific training, SSG training produced similar or greater improvements in most physical performance outcomes. Notably, the included evidence base was dominated by soccer cohorts (≈80% of participants), which may limit the generalizability of these findings to other youth team sports.

### 4.1 Cardiorespiratory endurance

Cardiorespiratory endurance is a key physical capacity for youth team-sport athletes during match play, as it supports sustained high-intensity running, rapid recovery, and continued engagement in repeated offensive and defensive actions. Its level is widely considered a physiological prerequisite for coping with changes in match tempo, repeated high-intensity efforts, and overall match load tolerance (Schmitz et al., 2018; Clemente et al., 2021). In this study, we quantified cardiorespiratory endurance using two outcome measures: maximal aerobic capacity and intermittent high-intensity endurance.

The meta-analysis included six studies examining maximal aerobic capacity. These studies showed that SSG training produced a moderate and statistically significant improvement in maximal aerobic capacity compared with control conditions (SMD = 0.78, p = 0.001), but between-study heterogeneity was moderate. The meta-analysis included four studies examining intermittent high-intensity endurance. These studies showed that SSG training produced a moderate and statistically significant improvement in intermittent high-intensity endurance compared with controls (SMD = 1.05, p< 0.001), and study-to-study consistency was high.

These findings are consistent with the results reported by Clemente et al. (2023). That systematic review and meta-analysis, which included 20 studies, compared SSG training with running-based HIIT in youth and adult soccer players for both maximal aerobic capacity and intermittent high-intensity endurance. In between-group comparisons, SSG training and running-based HIIT produced similar effects on maximal aerobic capacity and intermittent high-intensity endurance. In within-group analyses, SSG training produced significant pre–post improvements in maximal aerobic capacity (Hedges’ g = 0.50, p = 0.004), and it also produced significant pre–post gains in intermittent high-intensity endurance (for example, Yo-Yo intermittent recovery test level 1; Hedges’ g = 0.70, p< 0.001). Earlier, Impellizzeri et al. (2006) reported in a randomized trial of male youth soccer players that SSG training and running aerobic interval training produced comparable adaptations in VO_2_max and intermittent endurance and that both methods could elicit high cardiopulmonary stress (90–95% HRmax). In contrast, Moran et al. (2019), in a meta-analysis of seven studies in male youth soccer players, found that SSG training produced effects on cardiorespiratory endurance similar to conventional endurance training (SMD = 0.04, p = 0.86).

From a physiological and training perspective, these findings reflect the capacity of youth team-sport athletes to maintain overall output and recovery rhythms under repeatedly changing match loads (Bangsbo, 1994). Team-sport competition typically features intermittent loads alternating between high- and lower-intensity activity, and athletes must switch frequently between intensities (Iaia and Bangsbo, 2010). Therefore, the cardiovascular system’s ability to regulate under dynamic loads is important for sustaining match participation and endurance performance. For youth team-sport athletes, the development of cardiorespiratory endurance depends substantially on repeated, safe exposure to high heart-rate zones and on the progressive improvement of heart-rate regulation and recovery (Bangsbo, 1994; Iaia and Bangsbo, 2010). SSG manipulates pitch dimensions and SSG formats, and controls effective training time as well as rest between sets and rest between reps, so it simulates the spatial limits, contact situations, and task goals of real matches. This design makes the temporal structure and intensity fluctuations of training more closely resemble the demands of match play. Prior work has shown that SSG training can provoke high cardiovascular stress during sessions and that SSG training sessions exhibit large within-session intensity swings; the instantaneous intensity can reach the high zones typical of traditional HIIT, while the overall load organization remains closer to match conditions (Hill-Haas et al., 2011). In youth populations, this match-like intermittent load pattern in SSG training helps increase actual heart-rate exposure and improve cardiovascular regulation, which in turn better supports the endurance demands of repeated high-intensity exchanges in matches.

In the composite cardiovascular endurance outcome, the effect of SSG training on maximal aerobic capacity showed substantial heterogeneity across studies (I² = 61.2%). Sensitivity analyses indicated that the pooled effect remained stable and that heterogeneity decreased markedly after excluding influential studies, suggesting reasonable stability of the overall finding. Nonetheless, the included trials differed in intervention duration, sessions per week, training formats, and pitch dimensions, and also varied in single-session structure (e.g., sets, reps, effective training time, and rest time). Assessment methods also differed across studies. Notably, some studies estimated VO_2_max from the 20-m multistage fitness test, and this indirect field test is more sensitive to training-related changes and more vulnerable to test conditions and execution factors; these features can affect the magnitude of effect size and between-study consistency (Zaharia et al., 2023). Therefore, the finding for maximal aerobic capacity requires further confirmation under more uniform intervention designs and measurement protocols.

By contrast, the SSG training effect on intermittent high-intensity endurance showed greater consistency across the included studies and lower between-study heterogeneity. Still, this outcome is currently supported by a small number of studies, and some trials included multiple experimental groups compared with a single control group, which reduces the number of independent samples and may affect the robustness of effect-size estimates (Wang et al., 2024; Li et al., 2025; Wen et al., 2025). Thus, although the existing evidence points to a relatively stable benefit of SSG training for intermittent high-intensity endurance, confirmation in more independent studies is needed.

Overall, SSG training elicited differential responses across cardiovascular endurance indicators. In general, SSG training showed more consistent improvements in intermittent high-intensity endurance, while improvements in maximal aerobic capacity were more sensitive to differences in training design and assessment methods. Given that youth athletes are in a phase of growth and training adaptation, with aerobic systems still maturing and marked individual variability in responsiveness, this pattern of divergent findings is plausible. In practice, SSG training appears well-suited to develop intermittent high-intensity endurance that is relevant to match play, whereas systematic increases in maximal aerobic capacity may depend more on specific intervention design, task constraints, and alignment with the developmental stage of the athletes. The certainty of evidence was moderate for intermittent high-intensity endurance and low for maximal aerobic capacity; therefore, conclusions regarding maximal aerobic capacity should be interpreted more cautiously.

### 4.2 Linear sprint performance

Linear sprint performance is a fundamental physical capacity for youth team-sport athletes during competition, as it supports rapid displacement, the creation of defensive space, and key offensive and defensive transitions, and is closely related to decisive high-speed running actions and spatial contest behaviors during match play (Hopkins et al., 2009; Faude et al., 2012; Haugen and Buchheit, 2016; Guerrero et al., 2024; Neag et al., 2025).

When findings from previous systematic reviews and meta-analyses are considered together, the effects of SSG training on linear sprint performance remain inconclusive. Some evidence suggests that SSG training may preferentially benefit the sprint acceleration phase. Hammami et al. (2018), in a systematic review and meta-analysis including ten studies, reported that among team-sport athletes from soccer, handball, rugby, and basketball, SSG training produced a moderate and statistically significant improvement in short-distance linear sprint performance (10 m and 20 m) when compared with conventional training (SMD = −0.89, p< 0.05), suggesting that this training format may be more favorable for the development of sprint acceleration. However, results vary substantially across control conditions and study populations. Trotta et al., (2025), in a systematic review and meta-analysis including three studies, found that in female soccer players, SSG training did not result in statistically significant improvements in linear sprint performance (10 m, 20 m, and 30 m) when compared with running-based high-intensity interval training (HIIT) (SMD = −0.33, p = 0.316). Similarly, Kunz et al. (2019), in a systematic review and meta-analysis including nine studies, reported that in adolescent male soccer players, the effects of SSG training on linear sprint performance (5–40 m), relative to running-based HIIT, were generally trivial to small, and no practically meaningful improvement was observed.

To explain these inconsistent findings, several systematic reviews have discussed potential mechanisms related to training context and stimulus characteristics. Bujalance-Moreno et al., (2019) noted in their systematic review that, due to reduced pitch size and limited player numbers, SSG training often fails to sufficiently induce high-speed linear running stimuli, which may restrict the development of maximal sprint speed. Clemente et al., (2021) reported similar observations and suggested that the long-term adaptations of sprint speed following SSG training are unstable, with insufficient exposure to high-speed linear running being a likely contributing factor. Such inconsistencies are also evident across different sports. Li et al. (2024), in a systematic review focusing on youth basketball players, reported substantial variability in the effects of SSG training on linear sprint performance, with several studies showing no significant improvement.

Based on these findings, the present study distinguished sprint acceleration from maximal sprint speed within linear sprint performance and examined their responses to SSG training separately. The results of this meta-analysis indicate that, compared with conventional sport-specific training, SSG training produced a small but statistically significant improvement in sprint acceleration among youth team-sport athletes (SMD = −0.55, p = 0.019). However, moderate between-study heterogeneity was observed (I² = 59.8%). In contrast, maximal sprint speed showed a slight improvement trend that did not reach statistical significance (SMD = −0.30, p = 0.142), and heterogeneity remained moderate (I² = 43.6%). The contextual characteristics of SSG may explain this difference. Under conditions of restricted playing space and frequent offensive and defensive transitions, athletes repeatedly perform rapid starts and short-distance accelerations. However, they often need to decelerate, change direction, or engage in duels before reaching maximal sprint speed, limiting exposure to maximal sprint speed–specific stimuli.

From a kinematic and neuromuscular perspective, linear sprint performance comprises an acceleration phase and a maximal speed phase, which are governed by distinct mechanical and neuromuscular determinants (Hunter et al., 2005; Morin et al., 2012). Previous studies have shown that sprint acceleration mainly depends on the ability to generate high horizontal propulsive forces within short ground contact times, and performance during this phase is strongly influenced by neuromuscular activation efficiency, force orientation, and horizontal ground reaction force control (Hunter et al., 2005; Morin et al., 2012). In contrast, maximal sprint speed typically requires longer uninterrupted acceleration distances and sufficient exposure to high-speed linear running. Its performance is more closely related to the coordination of stride length and stride frequency, sprinting technique at high speed, and tendon elastic properties (Mero et al., 1992; Morin et al., 2012; Haugen and Buchheit, 2016). In team-sport training and competition contexts, especially among youth, athletes often decelerate, change direction, or engage in contact before reaching maximal speed. As a result, under SSG training conditions, mechanical and neuromuscular outputs related to sprint acceleration are more frequently stimulated. In contrast, the conditions required for maximal sprint speed development are relatively limited.

Sensitivity analyses showed that, after removing individual influential studies, between-study heterogeneity for both outcomes was substantially reduced, while the direction and statistical significance of the pooled effects remained unchanged, supporting the robustness of the findings. For sprint acceleration, the moderate heterogeneity was mainly attributable to the inclusion of the limited ball touch (LBT) condition in the study by Wen et al. (2025). This condition markedly increased short-distance start and acceleration exposure by strengthening task constraints, resulting in a higher training stimulus intensity than in most conventional SSG formats. After this condition was removed, heterogeneity was clearly reduced, which suggests that task constraint intensity may partially modulate sprint acceleration–related adaptations. In contrast, for maximal sprint speed, although the sensitivity analysis similarly reduced heterogeneity and the overall conclusion remained unchanged, no consistent moderating factor was identified, so no further mechanistic decomposition was conducted.

Overall, these findings indicate that SSG training is more effective for improving sprint acceleration, whereas their effects on maximal sprint speed appear limited. Given that the neuromuscular system and speed-related abilities of youth athletes are still developing, and that the formation of maximal sprint speed relies more strongly on sprint-specific technique, strength level, and sufficient exposure to high-speed running, these results appear reasonable. In training practice, SSG training can serve as a primary method for developing match-related short-distance acceleration. In contrast, its capacity to enhance maximal sprint speed is likely constrained by the training context and task constraints. The certainty of evidence for linear sprint performance outcomes (sprint acceleration and maximal sprint speed) was low (GRADE), so the pooled effects should be interpreted cautiously.

### 4.3 Change-of-direction ability

Change-of-direction ability is a fundamental physical capacity for youth team-sport athletes when they perform rapid deceleration, change direction, and re-accelerate during competition. This ability is closely linked to one-on-one confrontations, defensive actions, and spatial contests in decisive match situations (Pérez-Ifrán et al., 2023; Neag et al., 2024).

The meta-analysis of change-of-direction ability included seven studies. The pooled results showed that, compared with control conditions, SSG training produced a moderate and statistically significant improvement in change-of-direction ability (SMD = −0.85, p = 0.001), with moderate heterogeneity across studies (I² = 63.8%). Sensitivity analysis showed that the pooled effect remained stable after stepwise removal of individual studies, indicating that the overall finding was not driven by a single study.

These findings are consistent with the systematic review and meta-analysis by Trotta et al., (2025) in female football players. In that work, the authors reported that, compared with running-based high-intensity interval training, SSG training showed a small but statistically significant between-group advantage in change-of-direction ability, and a moderate and significant improvement was also observed within the SSG training group. At the same time, the authors noted that these results were based on a limited sample and therefore required cautious interpretation. Similar evidence was reported by Hammami et al. (2018), who conducted a systematic review and meta-analysis including seven studies across multiple team sports and found that SSG training produced a large and statistically significant improvement in change-of-direction ability (SMD = −1.49, p< 0.05), which is directionally consistent with the present findings.

However, conclusions from previous systematic reviews and meta-analyses on the effects of SSG training on change-of-direction ability have not been entirely consistent. In contrast, Clemente et al. (2021b) reported in their systematic review and meta-analysis that, in football players, neither SSG training alone nor SSG training combined with running-based training showed stable, consistent advantages for neuromuscular outcomes, such as change-of-direction ability. Likewise, a systematic review and meta-comparison by Kunz et al. (2019) identified only one study reporting change-of-direction outcomes in adolescent male football players. They did not perform a pooled analysis for this variable, indicating that the available evidence at that time was still limited. This lack of consistency across earlier studies obscured the true training effect of SSG on change-of-direction ability. In this context, the present study, by integrating additional evidence and increasing statistical power, demonstrates that SSG training can produce a moderate improvement in this outcome, with practical relevance.

From a physiological and neuromuscular control perspective, change-of-direction ability mainly depends on eccentric muscle force control during rapid deceleration and the athlete’s ability to re-accelerate immediately afterward, which places high demands on coordinated control and force regulation of the lower-limb muscles under high mechanical load (Sheppard and Young, 2006). In youth team-sport athletes, both training and competition commonly require frequent sudden stops, direction changes, and re-acceleration (Gaamouri et al., 2023; Konefał et al., 2023; Cao et al., 2024; Hammami et al., 2024). Within SSG settings, limited playing space and frequent offensive–defensive transitions repeatedly expose athletes to short-distance stopping, direction changes, and re-acceleration tasks. This high-frequency directional load closely matches how change-of-direction ability is actually used in competition (Hill-Haas et al., 2011; Clemente et al., 2021).

Taken together, the results of this section indicate that SSG training can lead to a practically meaningful improvement in change-of-direction ability in youth team-sport athletes. The magnitude of the training effect varied across studies, which may be partly explained by the fact that neuromuscular control and movement coordination are still developing during adolescence. This also suggests that exposure characteristics within the training design can influence outcomes. Although the present study increased statistical power by combining a larger body of evidence, these conclusions still require confirmation through additional high-quality studies. The certainty of evidence for change-of-direction ability was low, so the pooled effect should be interpreted cautiously.

### 4.4 Lower-limb explosive power

Lower-limb explosive power is a fundamental physical capacity for youth team-sport athletes and directly affects the quality of many competition-related jumping actions.

The results of this meta-analysis indicate that, compared with conventional sport-specific training, SSG training produced a moderate and statistically significant improvement in lower-limb explosive power (SMD = 0.60, p = 0.001). Between-study heterogeneity was low (I² = 0.0%).

The present findings are broadly consistent with earlier reviews. Kunz et al. (2019) and Clemente et al. (2021a) reported that, compared with running-based HIIT, SSG training did not produce a statistically significant between-group difference in vertical jump performance, which may reflect similar overall training intensity and relatively high baseline fitness among participants. Trotta et al., (2025) found that both training modes produced within-group gains in vertical jump performance. However, the between-group comparison was not significant, likely due to small sample size, participants’ training backgrounds, and the fact that vertical jump was not the primary training target (ES = 0.29, p = 0.126). By contrast, Hammami et al. (2018), in a meta-analysis of eight studies across multiple team sports, reported a small-to-moderate and statistically significant effect of SSG training on lower-limb explosive power (SMD = 0.68, p< 0.05), which is directionally consistent with the present findings. The current study builds on these reports by focusing specifically on youth team-sport athletes and by providing more targeted quantitative evidence for this population.

From a biomechanical perspective, lower-limb explosive power mainly reflects the ability to produce high rates of force within very short time frames, with these actions commonly exhibiting a deceleration–acceleration pattern that is closely related to the stretch–shortening cycle (Komi, 2003; Turner and Jeffreys, 2010; Maffiuletti et al., 2016; Chen et al., 2023). In training and competition, explosive lower-limb movements in youth team-sport athletes typically take the form of short-duration, high-intensity vertical or near-vertical efforts, such as rapid and repeated jumping, and these actions are often embedded within sequences of gameplay (Komi, 2003; Davids, 2012).

During SSG, athletes frequently perform jumping and explosive lower-limb actions in constrained spaces and dynamically changing situations, and the kinematic characteristics of these actions closely match those of lower-limb explosive power in team-sport competition (Davids et al., 2013; Clemente et al., 2021). For this reason, repeated implementation of explosive lower-limb actions in representative SSG scenarios may provide functionally relevant neuromuscular stimulation that supports adaptations in explosive performance. SSG intervention programs aimed at improving lower-limb explosive power varied in duration and frequency, and only a few studies reported systematic pre-post assessments of this capacity (Chaouachi et al., 2014; Wen et al., 2024; Xu et al., 2024; Majkić et al., 2025; Yang et al., 2025). Therefore, although the pooled effect showed high consistency, the evidence base remains limited, and the conclusions should be confirmed in larger, more diverse training programs and samples.

In practice, SSG training can deliver continuous, non-targeted stimuli for lower-limb explosive power in sport-specific contexts, and it is suitable as a supplementary method for developing explosive ability during youth development. If the training goal is to maximize vertical jump performance or peak explosive power, coaches should combine SSG training with targeted explosive-strength or jump training (e.g., plyometric training) to provide a more direct training stimulus (Chen et al., 2023). The certainty of evidence for lower-limb explosive power was moderate, supporting a more confident interpretation of the pooled effect.

## 5 Practical implications

A synthesis of previous systematic reviews and meta-analyses indicates that most research on SSG training has focused on adult athletes or on single sports. In contrast, systematic evidence on multidimensional physical adaptations in youth team-sport athletes remains limited. In this context, the present study systematically identified and included 15 relevant studies, quantitatively examined the effects of SSG training on physical performance in youth team-sport athletes and, based on the results, proposes the following practical implications.

First, SSG training in youth team-sport athletes shows relatively consistent improvements in intermittent high-intensity endurance and lower-limb explosive power. In addition, positive trends were also observed for maximal aerobic capacity, sprint acceleration, and change-of-direction ability, although the magnitude of these effects varied across studies. In contrast, no clear training advantage was observed for maximal sprint speed.

Second, based on the evidence from the present study, SSG training can be integrated into physical conditioning programs for youth team-sport athletes, supporting the development of multiple match-related physical performance capacities.

Third, for the optimal development of lower-limb explosive power, change-of-direction ability, and maximal sprint speed, SSG training appears more suitable as a complementary training method. In this context, combining SSG training with targeted strength training, plyometric training, and sport-specific sprint training is recommended to provide more direct and specific training stimuli.

Overall, in practical training settings, the present findings support the flexible integration of SSG training into routine training schedules while accounting for variations in training frequency and intervention duration. Such flexibility may help promote match-related physical performance in youth team-sport athletes.

## 6 Limitations

This study has several limitations.

Firstly, although this study systematically integrated the available randomized controlled trials, the number of included studies was relatively limited. The sample sizes used in meta-analyses of some physical performance outcomes were small, and subgroup analyses did not reveal significant moderating effects, limiting further assessment of the impact of different population characteristics or training variables.

Secondly, moderate between-study heterogeneity was observed in more than half of the included studies for the primary physical performance outcomes. Such heterogeneity may arise from variations in participant characteristics, intervention duration, training frequency, training protocols, and measurement approaches, which limited the comparability of results across studies.

Thirdly, due to limited literature, this study did not incorporate the seasonal timing of the intervention and the physiological maturity status of youth athletes into the systematic analysis. Therefore, this study was unable to assess potential differences in the effectiveness of SSG training across different training stages and maturity levels.

Finally, the included studies mainly focused on football players (≈80%), while the proportions of basketball and handball players were relatively low. Although no language restrictions were applied at the search stage, screening and full-text assessment were conducted in English, which may have introduced language bias and led to omission of relevant non-English studies. Differences in game rhythm and action characteristics across sports may limit the generalizability of research conclusions at the team-sport level.

## 7 Conclusion

The evidence from this systematic review and meta-analysis indicates that SSG training generally has positive effects on the physical performance of youth team-sport athletes. Specifically, SSG training significantly improves several physical performance indicators closely related to competition scenarios, including intermittent high-intensity endurance, sprint acceleration, lower-limb explosive power, and change-of-direction ability, with positive effects also observed for maximal aerobic capacity.

It is worth noting that although sprint acceleration and maximal sprint speed are often classified together as linear sprint performance, the training effects differ between the two, with greater improvements in sprint acceleration and limited effects on maximal sprint speed.

Furthermore, some physical outcomes (e.g., maximal aerobic capacity, sprint acceleration, and change-of-direction ability) exhibit moderate heterogeneity across studies. However, the results of the sensitivity analyses indicate that the overall conclusion is relatively robust.

Subgroup analyses further indicate that, under the current evidence base, the observed training effects were not significantly moderated by age, competitive level, total training sessions, or effective training time, with SSG training demonstrating generally positive effects on physical performance across these settings, thereby supporting its integration into routine youth training programs.

Future studies should aim to refine SSG training protocols and clarify how specific training configurations influence maximal sprint speed and other match-related performance outcomes in youth team-sport athletes. Given the predominance of soccer samples (≈80%), these conclusions should be generalized to other youth team-sport populations with caution.

## Data Availability

The original contributions presented in the study are included in the article/**Supplementary Material**. Further inquiries can be directed to the corresponding author/s.
