# Supplementary material for: Effects of small-sided games training on physical performance in youth team-sport athletes: a systematic review and meta-analysis
Source: Front Physiol. 2026 Apr 7;17:1803471. doi: 10.3389/fphys.2026.1803471 (PMC13095612; doi:10.3389/fphys.2026.1803471)
Supplement: Supplementary file 1 [file Table1.docx]

Supplementary Table 1. Meta-subgroup analysis results.

| **Outcome** | **Subgroup factor** | **Subgroup** | **No. of studies (k)** | **Pooled SMD (95% CI)** | **I² (%)** | **P for subgroup difference** |
| --- | --- | --- | --- | --- | --- | --- |
| Maximal aerobic capacity | Competitive level | Tier 2 | 3 | 0.81 (0.41, 1.20) | 0.0 | 0.926 |
|  |  | Tier 3 | 3 | 0.75 (–0.40, 1.90) | 84.3 |  |
|  | Total training sessions | >17 | 3 | 0.41 (–0.13, 0.96) | 39.4 | 0.066 |
|  |  | ≤17 | 3 | 1.16 (0.58, 1.75) | 46.8 |  |
|  | Effective training time | ≤15.5 min | 3 | 1.11 (0.43, 1.79) | 55.9 | 0.176 |
|  |  | >15.5 min | 3 | 0.49 (–0.08, 1.07) | 51.6 |  |
|  | Age | ≤16.9 years | 3 | 0.74 (0.32, 1.15) | 0.0 | 0.856 |
|  |  | >16.9 years | 3 | 0.84 (–0.22, 1.91) | 84.2 |  |
| Sprint acceleration | Competitive level | Tier 2 | 3 | –1.01 (–2.45, 0.43) | 86.9 | 0.410 |
|  |  | Tier 3 | 4 | –0.38 (–0.74, 0.3) | 0.0 |  |
|  | Total training sessions | >16 | 3 | –0.94 (–1.96, 0.08) | 80.5 | 0.252 |
|  |  | ≤16 | 4 | –0.30 (–0.69, 0.08) | 0.0 |  |
|  | Effective training time | >13 min | 3 | –1.05 (–1.95, 0.16) | 71.5 | 0.082 |
|  |  | ≤13 min | 4 | –0.20 (–0.56, 0.17) | 0.0 |  |
|  | Age | ≤16.1 years | 4 | –0.13 (–0.53, 0.27) | 0.0 | 0.054 |
|  |  | >16.1 years | 3 | –1.05 (–1.89, –0.20) | 72.9 |  |
| Maximal sprint speed | Competitive level | Tier 2 | 4 | –0.47 (–1.01, 0.06) | 55.2 | 0.322 |
|  |  | Tier 3 | 3 | –0.06 (–0.54, 0.42) | 62.3 |  |
|  | Effective training time | >12.7 min | 3 | –0.08 (–0.50, 0.33) | 0.0 | 0.295 |
|  |  | ≤12.7 min | 3 | –0.58 (–1.41, 0.25) | 71.8 |  |
|  | Age | >15.9 years | 3 | –0.51 (–1.16, 0.14) | 62.3 | 0.242 |
|  |  | ≤15.9 years | 3 | –0.0 (–0.49, 0.42) | 0.0 |  |
| Change-of-direction ability | Total training sessions | >16 | 3 | –1.17 (–2.04, −0.29) | 61 | 0.375 |
|  |  | ≤16 | 4 | –0.66 (–1.37, 0.06) | 70.6 |  |
|  | Effective training time | >13 min | 3 | –1.03 (–2.05, –0.01) | 71.8 | 0.665 |
|  |  | ≤13 min | 4 | –0.76 (–1.45, –0.07) | 68.4 |  |
